# Healthy life extension: Geroscience’s north star

**DOI:** 10.18632/aging.206359

**Published:** 2026-03-09

**Authors:** David A. Barzilai

**Affiliations:** 1 Geneva College of Longevity Science, Geneva 1204, Switzerland; 2 Healthspan Coaching LLC, Barzilai Longevity Consulting, Boston, MA 02111, USA; 3 Harvard Medical School, Boston, MA 02115, USA

**Keywords:** geroscience, longevity, healthspan, longevity medicine, healthy life extension

Mikhail Blagosklonny was right to say out loud: the goal of geroscience is life extension. Not “vitality” or a polite euphemism for better late-life care, but life extension [1]. He also insisted on disciplined evidence: if we claim we are modifying aging, we should demand hard outcomes in mammals rather than an endless parade of biomarkers.

Where I would extend his argument, as a longevity physician, is: the field must stop treating “lifespan vs. healthspan” as a fork in the road. In medicine, and in the lives our patients actually live, they are not competitors. The only mission that is both scientifically coherent and clinically meaningful is healthy life extension: more years in full health.

## The false choice that keeps the field small

The “healthspan, not lifespan” framing makes geroscience sound as though it is not about longevity, when longevity is what emerges from delaying the biology that drives multimorbidity.

World Health Organization (WHO) data show that from 2000 to 2019, life expectancy increased more than healthy life expectancy, meaning we added years lived with disease or disability [2]. A cross-national analysis quantified the global “healthspan to lifespan gap” at approximately 9.6 years [3]. Modern systems deliver more years, but not more good years.

That is precisely why geroscience must be more ambitious. We should treat healthy life extension as the goal and define success as health-adjusted longevity: extending lifespan while proportionally expanding function, resilience, and independence.

## A better target: health-adjusted survival

Geroscience should be judged by health-adjusted life extension, as measured by Health-Adjusted Life Expectancy (HALE), the average years lived in good health, and Quality-Adjusted Life Years (QALYs), which weight survival by functional status [4]. A year of life is not experienced uniformly; it is experienced through capacity, including mobility, cognition, freedom from pain, and the ability to work, love, and choose. The goal is not merely adding years, but adding years worth living.

The right rebuttal to “lifespan versus healthspan” is not picking sides. It is replacing the debate with a clearer objective: longer lives, in high function.

## Blagosklonny’s disciplined provocation

Blagosklonny’s insistence on lifespan evidence was field-building: a protest against a literature that celebrates “healthspan” using endpoints so elastic that they cannot fail.

Yet clinical reality forces a second truth: human trials cannot wait for death as the primary endpoint, and patients will not wait for science to become convenient. The practical bridge is to demand the right kinds of rigor, namely replicable lifespan effects in mammals where feasible, paired with human endpoints that map to health-adjusted survival, including delayed multimorbidity, frailty, function, and immune resilience.

The geroscience framework explicitly targets aging biology to influence multiple chronic diseases. Rapamycin’s lifespan extension in genetically heterogeneous mice remains a landmark, demonstrating that modulation of a conserved pathway can extend both median and maximal lifespan, even when treatment is initiated late [5]. Short-term mechanistic target of rapamycin (mTOR) inhibition boosted influenza vaccine responses in older adults in a randomized trial, offering an early signal that “aging biology” can be clinically legible on short timelines, even if broader replication is still pending [6].

## The physician’s perspective

In clinic, the “healthspan-only” posture breaks down immediately. Patients ask for time: time with family, to remain independent, and to keep thinking, creating, and living on their own terms. They fear the last decade of life, marked by frailty, falls, and cognitive decline, often as much as they fear death itself.

Frailty and function are not soft outcomes. They are among the most clinically predictive aging phenotypes, tied to hospitalization, disability, and mortality. They should sit alongside survival in our outcome hierarchy, not as consolation prizes but as the quality term in the longevity equation.

## The missing ingredient: a moonshot with real resources

If we agree that the goal is healthy life extension, incrementalism becomes a choice rather than a constraint. Consider the balance sheet: within the National Institute on Aging (NIA) budget, the Division of Aging Biology is funded at roughly $346 million, whereas neuroscience-related research is funded in the billions [7]. We have not resourced basic aging biology in proportion to its theoretical leverage: the possibility of delaying many diseases at once.

This is not a call to rob disease programs. It is a call to stop pretending a civilization-scale problem can be solved with niche-scale funding.

## A closing pledge

Blagosklonny’s legacy is that he demanded honesty about the goal and discipline about reaching it. Let us honor that by stating the mission without flinching:

Geroscience is for healthy life extension. We should stop pretending that lifespan and healthspan compete. The standard should be higher and simpler: extend lifespan by extending health, measured in HALE and QALYs, demonstrated through rigorous translation, and accelerated by a true moonshot in aging biology.

That is not a compromise. It is the completion of a single idea: more life, more life in full.

## References

[1] Blagosklonny MV. Oncotarget. 2021; 12:131–44. 10.18632/oncotarget.27882 33613842 PMC7869575

[2] Global health estimates Life expectancy and healthy life expectancy. https://www.who.int/data/gho/data/themes/mortality-and-global-health-estimates/ghe-life-expectancy-and-healthy-life-expectancy.

[3] Garmany A, et al. JAMA Netw Open. 2024; 7:e2450241. 10.1001/jamanetworkopen.2024.50241 39661386 PMC11635540

[4] Healthy life expectancy (HALE). https://www.who.int/data/gho/indicator-metadata-registry/imr-details/7752.

[5] Harrison DE, et al. Nature. 2009; 460:392–5. 10.1038/nature08221 19587680 PMC2786175

[6] Mannick JB, et al. Sci Transl Med. 2014; 6:268ra179. 10.1126/scitranslmed.3009892 25540326

[7] Fiscal Year 2025 Budget. National Institute on Aging. https://www.nia.nih.gov/about/budget/fiscal-year-2025-budget.

